# Recent Advances in the Application of Functionalized Lignin in Value-Added Polymeric Materials

**DOI:** 10.3390/polym12102277

**Published:** 2020-10-03

**Authors:** Yun-Yan Wang, Xianzhi Meng, Yunqiao Pu, Arthur J. Ragauskas

**Affiliations:** 1 Center for Renewable Carbon, Department of Forestry, Wildlife and Fisheries, University of Tennessee Institute of Agriculture, Knoxville, TN 37996, USA; ywang226@utk.edu; 2 Department of Chemical and Biomolecular Engineering, University of Tennessee, Knoxville, TN 37996, USA; xmeng5@utk.edu; 3 Joint Institute for Biological Science, Biosciences Division, Oak Ridge National Laboratory, Oak Ridge, TN 37831, USA; puy1@ornl.gov; 4 The Center for Bioenergy Innovation (CBI), Oak Ridge National Laboratory, Oak Ridge, TN 37831, USA

**Keywords:** lignin, functionalization, resins, thermoplastics, composites, copolymers, carbon fiber, adsorbents, nanoparticles

## Abstract

The quest for converting lignin into high-value products has been continuously pursued in the past few decades. In its native form, lignin is a group of heterogeneous polymers comprised of phenylpropanoids. The major commercial lignin streams, including Kraft lignin, lignosulfonates, soda lignin and organosolv lignin, are produced from industrial processes including the paper and pulping industry and emerging lignocellulosic biorefineries. Although lignin has been viewed as a low-cost and renewable feedstock to replace petroleum-based materials, its utilization in polymeric materials has been suppressed due to the low reactivity and inherent physicochemical properties of lignin. Hence, various lignin modification strategies have been developed to overcome these problems. Herein, we review recent progress made in the utilization of functionalized lignins in commodity polymers including thermoset resins, blends/composites, grafted functionalized copolymers and carbon fiber precursors. In the synthesis of thermoset resins such as polyurethane, phenol-formaldehyde and epoxy, they are covalently incorporated into the polymer matrix, and the discussion is focused on chemical modifications improving the reactivity of technical lignins. In blends/composites, functionalization of technical lignins is based upon tuning the intermolecular forces between polymer components. In addition, grafted functional polymers have expanded the utilization of lignin-based copolymers to biomedical materials and value-added additives. Different modification approaches have also been applied to facilitate the application of lignin as carbon fiber precursors, heavy metal adsorbents and nanoparticles. These emerging fields will create new opportunities in cost-effectively integrating the lignin valorization into lignocellulosic biorefineries.

## 1. Introduction

**Figure 1:**
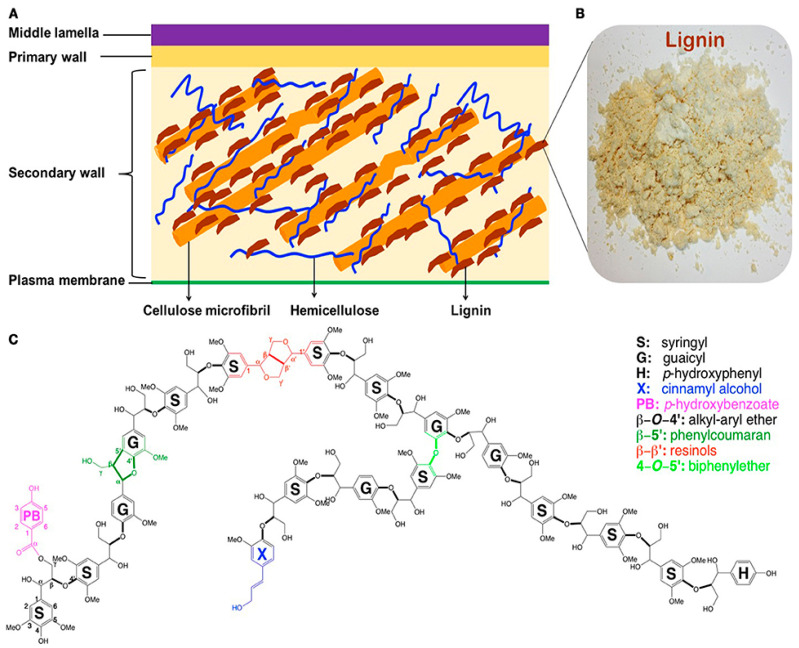
Chemical structure of lignin [4]. (**A**) Simplified structure of plant cell wall, (**B**) lignin isolated from hardwood poplar and (**C**) a representative structural model of hardwood lignin.

Lignin, the most abundant aromatic polymer on earth and the second most abundant terrestrial biobased organic polymer of any kind, is an amorphous, crosslinked and three-dimensional phenolic polymer. It accounts for 10 to 30% by weight in biomass, and its content varies among grasses, hardwoods and softwoods. Lignin imparts rigidity and strength to the plant cell wall and acts as a binding agent between wood cell walls [1]. Figure 1 shows a simplified structure of the plant cell wall and a representative structural model of hardwood lignin. Lignin mainly consists of three basic types of phenylpropane units as its monolignols, sinapyl, coniferyl and *p*-coumaryl alcohols, and the combinatorial free radical coupling of these three phenylpropanoid monomers gives rise to syringyl (S), guaiacyl (G) and p-hydroxyphenyl (H) lignin subunits (C9 units), respectively [2]. The coupling reaction forms mainly ether (C-O) interunit linkages which represent up to two-thirds of the total linkages, and it is generally favored at the β position of the monolignol species, thus resulting in the main linkages being β-O-4 (β-aryl ether) [3]. Meanwhile, a small amount of C-C linkages could be also formed at the β position during the oxidative radical–radical coupling reactions including β-β (resinol) and β-5 (phenylcoumaran) units. Native lignin also contains a very limited amount of 5–5 (biphenyl), 4-O-5 (diaryl ether) and β-1 (spirodienone) linkages. As shown in Figure 1, the lignin macromolecule contains various functional groups such as carbonyl, methoxy, carboxyl and hydroxyl groups.

A key challenge in understanding lignin chemistry and utilizing lignin for value-added polymeric materials is the inability to effectively isolate lignin from lignocellulosics in its native state, and the physicochemical features and composition of lignin strongly depend on the chemical process used for its extraction [5]. Currently, technical lignins are produced from four major processes, Kraft, sulfite, soda and organosolv, at industrial scale. Depending on the chemicals applied in the process, these technical lignins can be classified into sulfur-containing and sulfur-free. Each of them undergoes dramatic structural changes as a result of chemical treatment; thus, each is unique in terms of molecular weight, chemical structure, thermal properties and ultimate applications [6]. Currently, sulfite lignin generated by the sulfite process with ~1000 kt lignosulfonates per year has the largest valued-added application, with a wide range of industrial applications such as binders, emulsifiers, chemicals and dispersants [7]. In industrial sulfite pulping, wood chips are delignified using a sulfite or bisulfite salt under acidic conditions (pH 1.5−2.0 for the sulfite process and pH 4.0−5.0 for the bisulfite process) at 140–160 °C, and lignin undergoes sulfonation mainly at C_α_, cleavage of aryl ether bonds and condensation reactions [8]. The byproducts, lignosulfonates, are high-molecular-weight (15,000−50,000 g/mol), water-soluble polyelectrolytes with a high sulfur content (~5 wt%) [9]. The Kraft process, the most dominant process in the papermaking industry, treats wood chips in the aqueous mixture of sodium hydroxide and sodium sulfide solution at 150−180 °C for 2−3 h [10]. The cleavage of aryl ether bonds creates additional free phenolic groups; therefore, Kraft lignin is soluble in aqueous alkaline solution (pH > 10.5) and in some organic solvents such as dimethyl sulfoxide (DMSO) and dimethylformamide (DMF). During Kraft pulping, the nucleophilic attack of hydrosulfide ion on lignin retains some thiol groups; thus, Kraft lignin usually contains a small amount (<3%) of sulfur [10,11]. Sulfur-free lignin isolation processes present a much smaller portion (<2%) in terms of the annual production of commercial lignin [7]. Soda process is commonly applied to annual plants or agricultural residuals containing low lignin contents such as straw, bagasse, bamboo and flax [12]. Soda lignin is depolymerized and solubilized in aqueous sodium hydroxide solution at 150−180 °C. Similar to the Kraft process, soda lignin can be recovered by acidification, but it has higher purity and lower molecular weight (800−3000 g/mol) than Kraft lignin (1500−5000 g/mol). In the organosolv process, lignin is typically extracted from biomass with a mixture of a polar organic solvent and water under mild acidic conditions. Although it holds the smallest market share among the four major processes, it has been given intensive attention in the field of lignocellulosic biorefineries due to the eco-friendly conditions and easy recovery of organic solvents employed in organosolv processes. Novel organosolv processes adopting biomass-derived organic solvents such as γ-valerolactone [13], tetrahydrofuran [14], 2-methyltetrahydrofuran [15] and cyrene [16] have been developed to achieve a “closed-loop” biorefinery. In general, organosolv lignins have low molecular weight (500−5000 g/mol) with a narrow molecular weight distribution (1.5–2.5), and they exhibit good solubility in a wide range of organic solvents [9].

## 2. Covalent Incorporation of Functionalized Lignin

### 2.1. Polyurethanes

**Figure 2:**
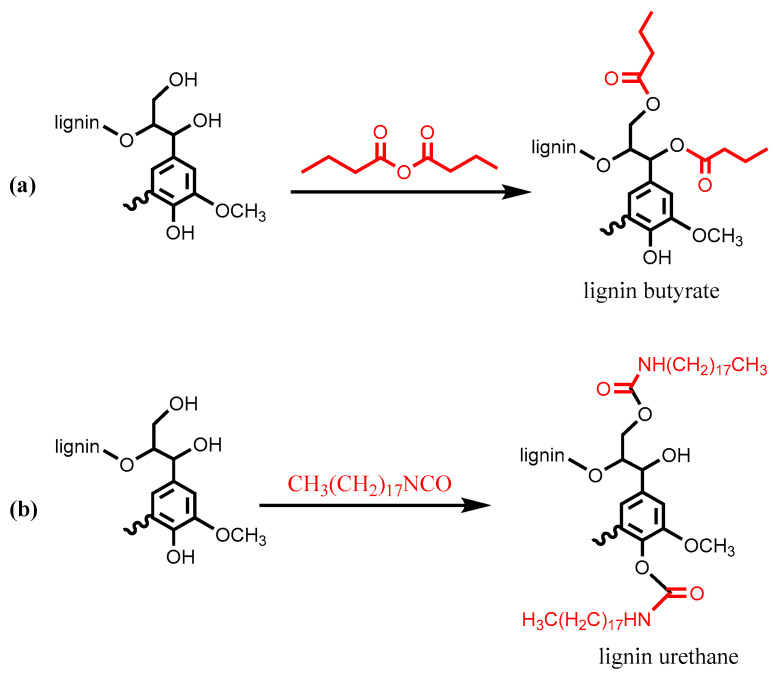
Synthesis of lignin butyrate (**a**) and lignin urethane (**b**). Adapted from [22] with permission from Elsevier © 2020.

Polyurethanes (PU) have been widely applied in flexible/rigid foams and non-porous materials such as coatings, adhesives, sealants and elastomers [17]. The basic PU chemistry involves reacting the isocyanate group (−N=C=O) with a hydroxyl group (−OH) to form a urethane bridge (−NHCO−O−) [18]. Owing to the multifunctionality of macromolecular lignin, the application of technical lignins in PU materials as replacements for petroleum-based polyols can be dated back to the 1960s, ~30 years after the discovery of PU [19,20]. Technical lignins which are covalently incorporated into the network are expected to perform as hard segments to enhance the physical strength of the PU materials [21]. Demethoxylation, amination, nitration, phenolation and oxypropylation/etherification are common approaches that have been used to improve the reactivity of lignin [9]. Zhang et al. derivatized a commercial lignin with octadecyl isocyanate and butyric anhydride to reduce the polarity contributed by hydroxyl groups in lignin macromolecules (Figure 2) [22]. These modified lignin samples were employed as fillers in the vegetable oil-based PU composites. It was found that lignin urethane was more compatible with the PU matrix than lignin butyrate, and up to 30 wt% lignin urethane was incorporated without deteriorating the mechanical properties of the final product. Jang et al. synthesized poly(ε-caprolactone) grafted lignin (PCL-*g*-lignin) via ring-opening polymerization of ε-caprolactone in the presence of organosolv lignin. The PCL-*g*-lignin and acrylic polyol were mixed with NCO-terminated polyethylene glycol to produce PU-based gel-coated films [23]. As the content of PCL-*g*-lignin increased to 35 wt%, the surface characteristics of the films, such as adhesion strength, abrasion and gloss, remained relatively constant, but the tensile strength and hardness of the films decreased, and elongation at break increased due to the reduction in crosslinking density.

**Figure 3:**
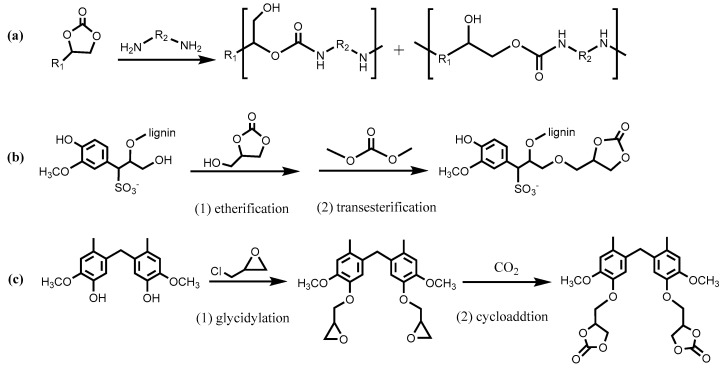
(**a**) Synthetic scheme of NIPU); (**b**) two-step synthesis of lignosulfonate-glycerol cyclic carbonate; (**c**) cyclic carbonate synthesized from CO_2_ fixation in epoxides [24,25].

The synthesis and degradation of conventional PU materials involve phosgene (used in synthesizing isocyanates such as toluene diisocyanate and methylene diphenyl diisocyanate) and hazardous isocyanate compounds. Recently, non-isocyanate PUs (NIPU) have been produced from lignin derivatives via polyaddition between cyclic carbonates and amines (Figure 3a) [24,25]. Mimini et al. prepared cyclic carbonate functionalized lignosulfonate via base-catalyzed two-step synthesis: etherification with glycerol carbonate, followed by transesterification with dimethyl carbonate (Figure 3b) [24]. Rubbery NIPU materials were produced by reacting the obtained lignosulfonate-glycerol cyclic carbonate with excessive 1,6-hexamethylenediamine at 70 °C. Functionalization with cyclic carbonate can be achieved alternatively via glycidylation of the hydroxyl group, followed by CO_2_ fixation (cycloaddition), as depicted in Figure 3c. Chen et al. functionalized lignin-based bisphenol via a CO_2_ fixation route and successfully polymerized it with diamines with 10 mol% organocatalyst, 1,5, 7-triazabicyclo[4,4,0]-dec-5-ene (TBD) [25].

### 2.2. Lignin-Phenol-Formaldehyde (LPF)

**Figure 4:**
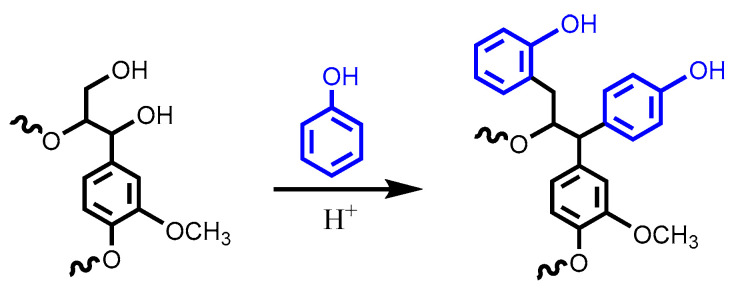
Acid-catalyzed phenolation of lignin. Reproduced from [28] with permission from American Chemical Society © 2020.

Phenol formaldehyde (PF) resins are synthesized via step-growth polymerization between phenol and formaldehyde. Nowadays, more than 50% of the PF resins produced in the U.S. are applied as wood adhesives for engineered wood products [26]. The phenolic structure of lignin makes it a renewable and inexpensive replacement for the petroleum-based phenol derived from benzene through the cumene hydroperoxide process [27]. In the synthesis of lignin-based PF resins, formaldehyde initially substitutes at the free ortho position of the lignin phenolic hydroxyl group. The formed hydroxymethyl group can further polymerize with another aromatic ring through a methylene or an ether bridge. Several strategies including methylolation, demethylation, phenolation, sulphonation and depolymerization have been designed to increase the reactivity of technical lignins for producing PF resins [27]. Phenolation can be conducted under both acidic and basic conditions, accompanied by depolymerization. Recently, Podschun et al. conducted H_2_SO_4_-catalyzed phenolation of organosolv, Kraft, sulfite, soda and hydrolysis lignin isolated from hardwood, softwood and annual plants [28]. Phenol substitution occurs on the aliphatic hydroxyl groups; therefore, the degree of phenolation was mainly affected by the number of aliphatic hydroxyl groups of the raw lignins (Figure 4). Phenolysis causes a reduction in the molecular weight of the phenolated product and also contributes to the degree of phenolation. Auto-catalyzed phenolation was achieved for sulfite lignin by ion exchange to proton form, and the degree of phenolation was improved vastly. Acid-catalyzed phenolation was inefficient for Kraft lignin due to the presence of the alkaline ash residue from pulping. The studies on phenolation under alkaline conditions demonstrated that the number of active sites on technical lignins and lignin-rich bioethanol fermentation residues increased effectively at mild temperatures (<100 °C), and the alkaline phenolation treatment can be integrated with the NaOH-catalyzed LPF resin adhesive synthesis without additional purification of the phenolated products [29,30,31]. Gan and Pan investigated NaOH-catalyzed phenolation of Kraft lignin at high temperatures (>100 °C) [32]. Lignin depolymerization was observed as side-chain cleavage was promoted by raising the reaction temperature or increasing phenol dosage, and Kraft lignin was degraded to 51% phenolated lignin segments and 49% small organic compounds after 2 h reaction at 160 °C with phenol/Kraft lignin = 3:1.

Li et al. compared the demethylation of soda lignin catalyzed by four sulfur-mediated reagents, S, NaSH, Na_2_SO_3_ and *n*-dodecyl mercaptan, in aqueous NaOH solution at 90 °C [33]. The demethylation reactions were purposely conducted at atmospheric pressure to lower the production cost of fast curing LFP at an industrial scale. Na_2_SO_3_ was shown to be the most effective in demethylating lignin. With 30% phenol replaced by demethylated lignin, the plywood bonded with LPF-Na_2_SO_3_ showed formaldehyde emission and bonding strength comparable to PF resin without lignin. Wang et al. prepared and characterized LPF adhesives with 60% phenol substituted with demethylated wheat straw alkali lignin [34]. The phenolic hydroxyl group of wheat straw alkali lignin was increased from 5.2% to 16% via demethylation catalyzed by a Lewis acid, iodocyclohexane, at 145 °C in *N, N*-dimethyl formamide (DMF).

### 2.3. Epoxy

Epoxy resins are known to be a class of prepolymers containing reactive epoxide (glycidyl) groups. Most commercial epoxy resins are polymerized by reacting bisphenol-A with epichlorohydrin. Catalyzed by NaOH, the grafting of epoxy groups occurs between the primary carbon of epichlorohydrin and the phenolic hydroxyl groups [27]. Two common approaches have been developed to covalently incorporate technical lignin into epoxy resins: direct epoxidation of lignin and pre-modification of lignin before epoxidation [27]. The poor solubility of lignin in most organic solvents and its low reactivity caused by steric hindrance constrain the application of directly glycidylated lignin in epoxy resin. Pre-modification of lignin usually aims to increase the number of reactive sites on the lignin aromatic ring. For example, Zhang et al. derived an industrial-grade organosolv lignin via demethylation, phenolation and hydroxymethylation to enhance lignin reactivity in epoxidation [35]. However, the improvements in the mechanical properties of the epoxy resins were not impressive compared with the ones made from raw lignin.

**Figure 5:**
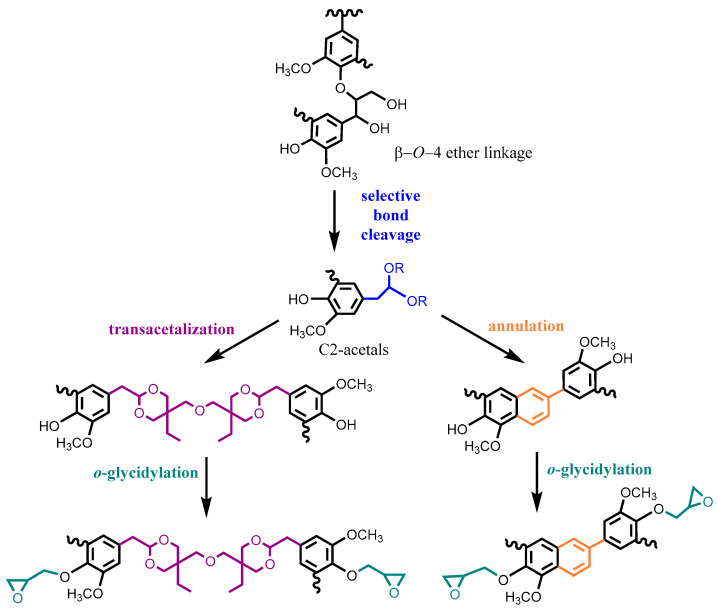
Synthetic scheme of lignin-based epoxy resin from selectively depolymerized lignin. Adapted from [36] with permission from The Royal Society of Chemistry © 2020.

**Figure 6:**
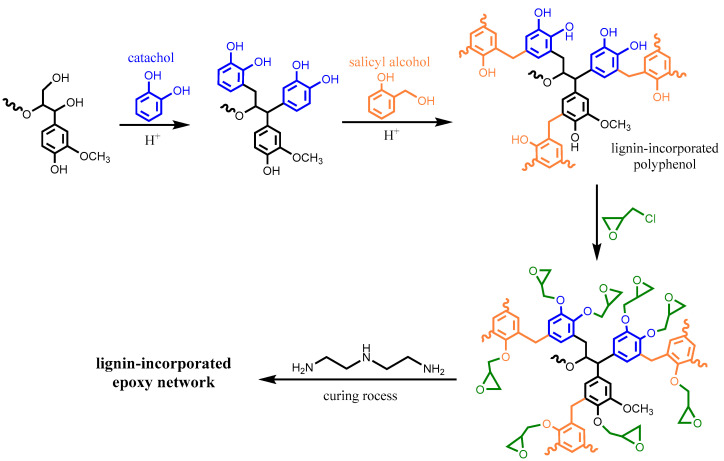
The synthetic route of liquid lignin-incorporated polyphenol from acid-catalyzed phenolation and formaldehyde-free oligomerization. Adapted from [38] with permission from American Chemical Society © 2020.

Multi-step derivatization of lignin prior to glycidylation usually involves a combination of demethylation, phenolation and depolymerization along with other modification methods. For example, Kaiho et al. degraded polymeric lignin through selective β-*O*-4 bond cleavage in the presence of methanol as a trapping reagent [36]. Before gylcidylation, two modification strategies were employed on the obtained low-molecular-weight C2-acetal lignin fragments, as illustrated in Figure 5: (1) transacetalization with tetraol di(trimethylolpropane) (DTMP) to form a flexible structure and (2) intramolecular annulation to form a rigid phenylnaphthalene structure. As expected, the structural design of the selectively depolymerized lignin was able to control the thermal and mechanical properties of the lignin-based epoxy resins to meet different requirements. For example, the epoxy-DTMP-lignin possessed a lower glass transition temperature that was close to epoxy-bisphenol A (95 °C); on the other hand, remarkable improvements (>50%) in flexural and impact strengths were achieved for epoxy using annulated lignin. To form a homogenous crosslinked epoxy network, glycidylated lignin pre-polymer was preferred to be a liquid. In the preparation of lignin-incorporated novolac polyphenols, Zhao et al. liquified the solid organosolv lignin by successive demethylation, phenolation, phenol-formaldehyde reaction and glycidylation, yet the lignin content was limited to 12 wt% due to the poor compatibility [37]. To solve this problem, the organosolv lignin was first phenolated with catechol to break down the lignin backbone and increase the number of hydroxyl groups, and subsequently, the catechol ortho site of the phenolated lignin was condensed with salicyl alcohol in water without using formaldehyde as the coupling agent (Figure 6) [38]. In the cured epoxy thermosets, the final lignin content was up to 19 wt% and total biomass content including catechol and salicyl alcohol derived from renewable sources reached 65–69 wt%.

**Figure 7:**
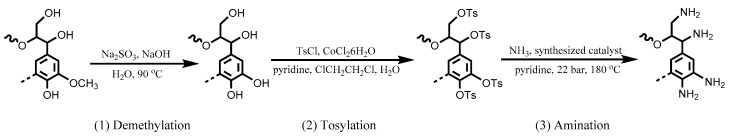
Direct primary amination of lignin. Adapted from [39].

Amines including aliphatic amine, aromatic amine and modified amines are common curing agents/hardeners reacting with epoxy resins to form crosslinked thermosets [27]. Amination that introduces amine groups to lignin aromatic rings provides an alternative method to blend modified lignin into epoxy and thermally cured. Nikafshar directly added primary amine groups to a spruce Kraft lignin via successive demethylation, tosylation and cobalt/copper-catalyzed amination (Figure 7) [39]. The primary aminated lignin was applied to crosslink the diglycidyl ether of bisphenol, and the authors concluded that mechanical performance was related to the bulky molecular structure of lignin, and the content of aminated lignin in the cured thermoset should not surpass ~13%. Mendis et al. functionalized a Kraft lignin preparation via a Mannich reaction that extended the lignin aromatic ring with primary and secondary amine groups using an additional methylene bridge derived from formaldehyde [40]. The Mannich amination improved the dispersion of lignin in the epoxy matrix, which led to comparable thermomechanical properties to neat epoxy.

Another trend for lignin-based curing agents is to add carboxylic acid groups to lignin molecules through esterification with anhydrides. Sun et al. functionalized the Kraft lignin with succinate anhydride in pyridine and then utilized the carboxylated lignin (up to 10 wt%) as a co-hardener in an epoxy-amine system [41]. Most of the chemical modification of lignin was carried out in the solvent, yet the conversion efficiency was affected by the solvation behavior of lignin. Guo et al. esterified a commercial Kraft lignin with succinate anhydride (SA) through a one-step solvent-free ball milling process [42]. In the presence of succinate anhydride, the molecular weight of succinylated Kraft lignin (SA-KL) was greatly reduced after ball milling, and the esterification conversion reached the maximum when the stoichiometric ratio of SA to KL hydroxyl was 1.8. In combination with a carboxyl-terminated copolymer of acrylonitrile and butadiene liquid rubber reducing the intrinsic brittleness brought by the lignin components, up to 38 wt% SA-KL was successfully incorporated into the aliphatic glycidyl ether modified bisphenol A/F epoxy as a curing agent.

### 2.4. Lignin-Based Copolymers

Lignin-based copolymers are usually synthesized via controlled polymerization including atom transfer radical polymerization (ATRP), reversible addition fragmentation chain transfer (RAFT) and ring-opening polymerization (ROP). The “graft from” method, in which the lignin macromolecules serve as a core unit and new polymer chains grow on the initiating sites, is considered as a cost-effective way to produce lignin-based copolymers at industrial scale [43]. The grafted polymers containing new functional groups expand the utilization of lignin further in biomedical nanofibers, thermoplastics and additives such as dispersants, flocculants and surfactants (Table 1) [44,45,46,47,48,49,50,51,52,53].

Recently, nanofibers of lignin-based copolymers were fabricated by electrospinning for exploring the biomedical applications of lignin [45,46,47]. Kai et al. prepared poly(methyl methacrylate) (PMMA)-grafted lignin copolymers via the ATRP method and produced a nanofibrous composite by blending the lignin-PMMA copolymers with poly(ε-caprolactone) (PCL). They found that the presence of PMMA could improve the miscibility of lignin in PCL by tuning the grafted PMMA chain length. Poly(ε-caprolactone-*co*-lactide) (PCLLA) and poly(3-hydroxybutyrate) (PHB) copolymers were grafted onto lignin via a solvent-free ROP approach, respectively [45,47]. The morphological studies showed that these polyester-grafted lignin copolymers exhibited excellent miscibility in the host polyester matrix. However, their impacts on the mechanical properties were quite different despite the fact that polyester/lignin copolymer blends were able to form beadless and uniform nanofibers. For example, lignin-PCLLA was capable of reinforcing PCL to a great extent, but it failed to show any encouraging improvements in the tensile properties of poly(L-lactic acid) (PLLA) [45]. Yet, the incorporation of lignin gave the polyester nanofibers some advantages, including high antioxidant activity and fast degradation rate in phosphate buffered saline. These polyester-grafted lignin nanofibers are noncytotoxic according to the biocompatibility assessments in vivo; therefore, they can be used in potential medical or healthcare products [45,47].

The industrial application of Kraft lignin is hindered by its low solubility in water. Kong et al. copolymerized Kraft lignin with acrylic acid in aqueous alkaline solution [44]. Under optimized conditions, the resulting Kraft lignin-PAA copolymer was water-soluble even at pH 4. Another water-soluble Kraft lignin-based copolymer, lignin-g-poly(acrylamide)-g-poly(diallyldimethyl-ammonium chloride) (lignin-PAM-PDADMAC), was found to be an effective flocculant for kaolin suspension in wastewater treatment [52]. A bio-based triple shape memory copolymer was prepared by crosslinking lignin hydroxyl groups with the terminal carbonyl groups on the hyperbranched poly(ester-amine-amide) (HBPEAA) [48]. It was reported that the first recovery of lignin-HBPEAA was related to the lignin-poly(ester-amine)-rich network and the second recovery was related to the lignin-poly(ester-amide)-rich network. Thus, the thermoresponsive behavior that was achieved through the combined effects of the glass transition of polyester copolymers, lignin, hydrogen bonding and crosslinking density can be adjusted by tuning the composition and synthetic approach of lignin-HBPEAA.

## 3. Functionalized Lignin-Based/Containing Blends, Composites

**Figure 8:**
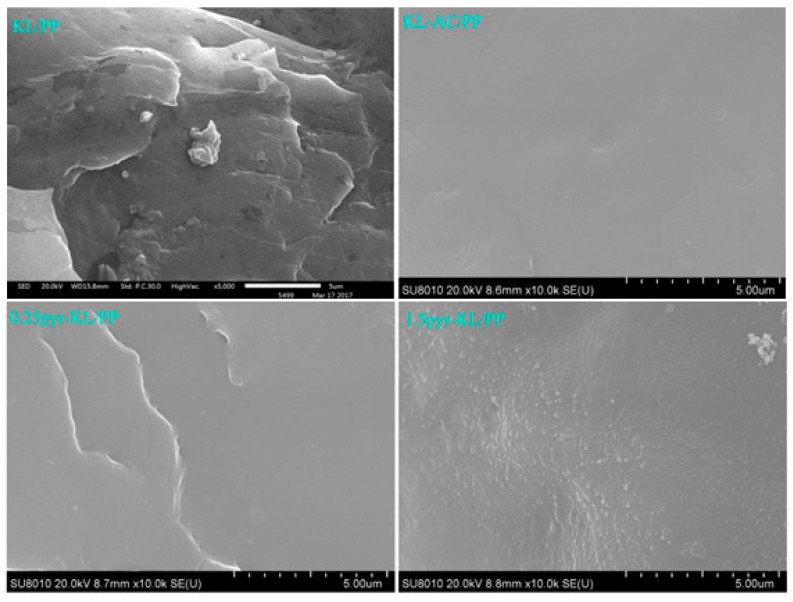
SEM micrographs of polypropylene blending with 0.5 wt% Kraft lignin and its derivatives. (AC: acetylated, 0.25 pyr-KL and 1.5 pry-KL contain 1.07 mmol/g and 3.07 phenolic-OH, respectively). Reprinted from [61] with permission from Elsevier © 2020.

The applications of lignin in crosslinked materials discussed above focus on enhancing the reactivity of lignin via single or multiple-step chemical modification. However, one of the most cost-effective ways to utilize technical lignin is to blend it with commodity polymers through melting mixing and numerous efforts have been devoted to investigating different lignin-polymer systems in the past few decades. Understanding the impact of material interactions on miscibility is essential for formulating a lignin-polymer system with desirable properties [54]. Pristine lignin by itself is an amorphous polymer constituted by a rigid aromatic backbone, polar chemical groups. The attractive intermolecular interactions between lignin components arising from hydrogen bonding and π−π electron correlation of the aromatic rings cause association/aggregation of lignin macromolecules in various organic solvents and aqueous solutions as well as in the polymer matrix [55,56,57]. When blending with other polymers, the poor miscibility and dispersion of lignin components could cause defects in the material matrix; therefore, rapid deterioration of mechanical properties was frequently observed when the content of lignin increased [58]. Lignin modification via alkylation and esterification are two common approaches applied to substitute the hydroxyl groups and consequently reduce lignin–lignin interactions [59]. Dehne et al. esterified five technical lignins of different plant species and isolation methods with acetic, propionic and butyric anhydride, respectively [60]. Unmodified and esterified technical lignins were blended with high-density polyethylene (PE) with a weight ratio of 1:1 to produce twenty formulations. Their systematic evaluation of the influence of esterification showed a progressive increase in both tensile and flexural strength with the increasing length of the ester carbon chain. In another study of modified lignin blending with nonpolar polymer, Ye et al. reported selective aminolysis of acetylated Kraft lignin (pyr-KL) to obtain high content of free phenolic groups, while most of the aliphatic ones were blocked in the acetate form [61]. Polypropylene (PP) blending with 0.5 wt% of pyr-KL exhibited anti-oxidation capacity 2.6 times higher than that of pure PP without weakening its mechanical properties. In fact, as indicated in the SEM micrographs (Figure 8), the miscibility between PP and softwood KL was tuned by the content of phenolic hydroxyl groups.

**Figure 9:**
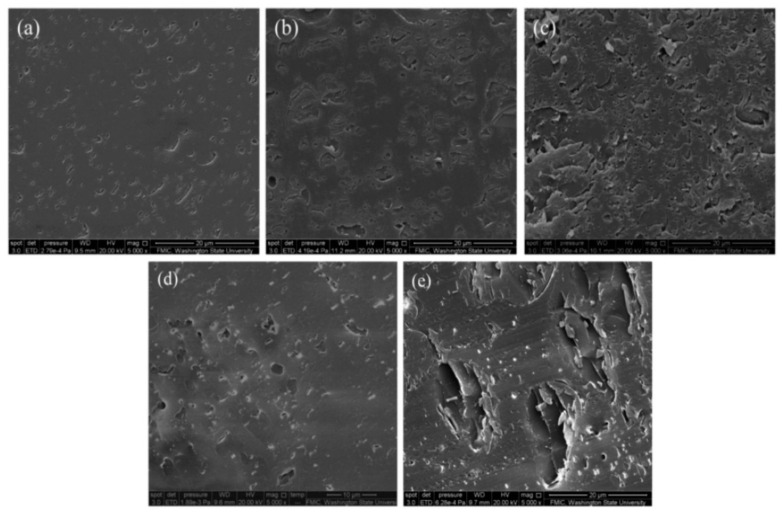
Cross-section of surface of PLA/organosolv lignin blends with and without mechanochemical oleation: (**a**) 30 wt%, (**b**) 50 wt%, (**c**) 70 wt% oleated organosolv lignin and (**d**) 30 wt%, (**e**) 50 wt% milled organosolv lignin. Reprinted from [64] with permission from Wiley © 2020.

Poly(lactic acid) (PLA) is a biodegradable thermoplastic polyester produced from agricultural waste. PLA has good melt processability and high strength, but it is weak in ductility and thermal stability [62]. Although homogeneous, single-phase morphology was reported for the blends of cellulolytic enzyme lignin and PLA, technical lignins tend to have poor miscibility with PLA [63]. Guo et al. mechanochemically esterified organosolv lignin with long-chain oleic acid (C_18_H_34_O_2_) by ball milling [64]. The dispersion pattern of lignin particles in the PLA matrix illustrated in Figure 9 indicated that oleation improved the compatibility of lignin with PLA. Its material strength decayed with the growing size of lignin particles, and the upper limit of oleated lignin incorporation in the PLA blend should not exceed 50%, since 70% oleated lignin retained only ~20% of the strength of neat PLA. Organosolv pine lignin was modified by butyric, isobutyric and crotonic anhydrides to replace the lignin hydroxyl groups with a four-carbon ester structure. It turned out that the presence of esterified lignin cause a reduction in tensile strength of 30% for all three PLA/lignin blends, but the ductility was obviously improved by lignin with saturated ester side chain, especially for lignin butyrate, of which the elongation at break reached 18.1% in comparison with 1.88% for the neat PLA [65]. Other simple modifications of lignin such as acetylation were also applied to improve the ductility and thermal properties of PLA/lignin. Gordobil et al. generated PLA composites using 0.5~20% lignin as a filler by extrusion at 200 °C [66]. With 0.5% and 5% acetylated Kraft lignin, the onset of thermal degradation was delayed by 70−80 °C. However, the tensile strength of PLA/acetylated Kraft lignin dropped dramatically, and the composite became brittle when the content of lignin increased beyond 10%. 

**Figure 10:**
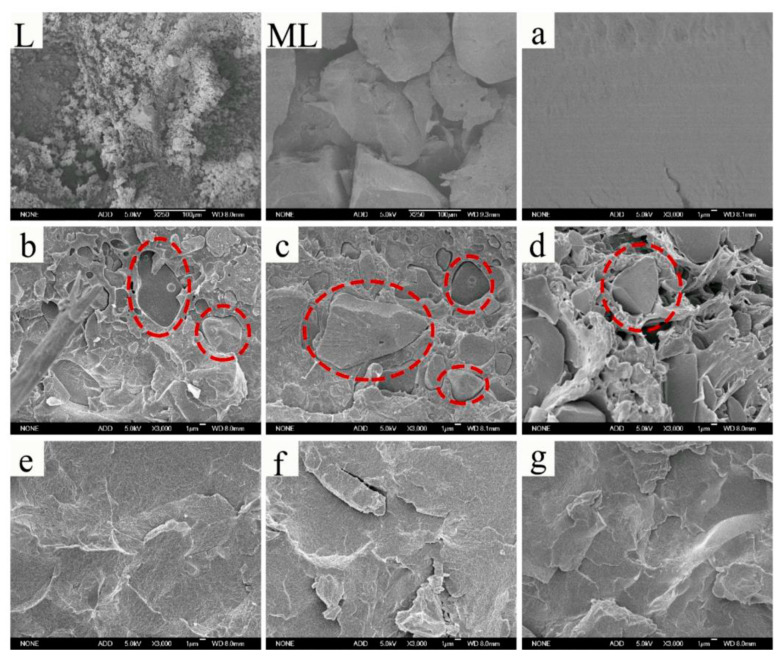
SEM micrographs of lignin (L), methylated lignin (ML) and surface morphologies of (**a**) neat PBTA film and PBTA/L (*w*/*w*) composite films: (**b**) 60/40, (**c**) 50/50, (**d**) 40/60 and PBTA/ML composite films (*w*/*w*): (**e**) 60/40, (**f**) 50/50, (**g**) 40/60. Reprinted from [67] with permission from American Chemical Society © 2020.

Xiong et al. fabricated high-performance composites with high lignin content (40–60 wt %) by melt blending poly(butylene adipate-co-terephthalate) (PBAT) with a low-molecular-weight eucalypt hydrothermal lignin preparation (M_w_ = 1580 g/mol) [67]. The compatibility of PBAT with lignin was assumed to be better than that of PLA due to π electron interactions between aromatic rings [63]. The SEM micrographs (Figure 10b–d) of the PBAT/lignin composites showed large particles formed by lignin self-aggregation. After methylation, the intermolecular interaction between lignin macromolecules was weakened, and lignin agglomerates seemed to disappear in the PBAT/methylated lignin composites even under the circumstance that lignin became the dominate component (60 wt% methylated lignin) in the composite films (Figure 10e–g) [67]. In fact, alkylation was proven to be an effective method to produce lignin-based thermoplastic blends [55,56,68]. Wang et al. demonstrated that both methylated lignosulfonate and ball-milled lignin, in the presence of suitable plasticizers, can be converted into polymeric materials containing > 80% lignin [69,70]. These lignin-based polymeric materials exhibited tensile strength surpassing polystyrene, yet their poor ductility implies that alkylation alone is not sufficient to overcome the inherent brittleness of lignin. 

## 4. Lignin as Carbon Fiber Precursors

**Figure 11:**
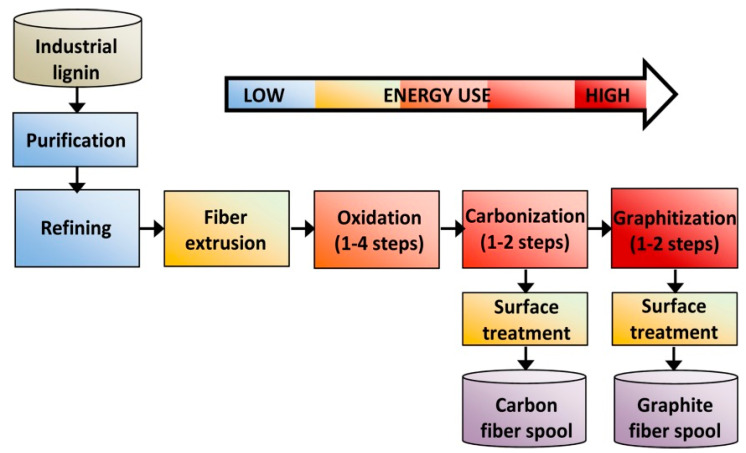
A schematic of carbon fiber production from an industrial lignin. Reprinted from [74] with permission from Wiley © 2020.

Carbon fibers are lightweight carbon materials with superior mechanical performance and are around 5–10 μm in diameter. They have been widely used in industries such as automotive, sporting goods, aerospace, wind energy, thermoplastic compounding, 3D printing and other structural applications due to their high tensile strength, high stiffness, high chemical resistance, high-temperature tolerance, and low weight and low thermal expansion [71]. Carbon fiber production consists of four major steps: lignin purification/refining, fiber spinning/extrusion, thermo-stabilization and carbonization (Figure 11) [72]. Different spinning techniques have been also developed including dry, wet, melt and electrospinning. Today, one of the most commonly used precursors to produce carbon fiber is petroleum-derived polymer polyacrylonitrile (PAN), and ~50% of the cost of carbon fiber belongs to the cost of the PAN precursor [73]. Because the conventional high-performance carbon fiber manufacturing process is very costly, the effective utilization of cost-effective alternatives such as lignin as carbon fiber precursors has been extensively investigated. Despite significant efforts being made in discovering lignin’s potential in carbon fiber production, the heterogeneity and poor mechanical performance of the final products still significantly hinders the commercialization process. For example, no pure lignin-based carbon fibers without the addition of PAN have been made to meet the mechanical standard (1.72 GPa tensile strength, 172 GPa tensile modulus) required for the automotive industry to date [74]. As an alternative approach, recent studies have been focused on the blend processing of PAN with lignin, which takes advantage of the good spinnability of PAN and the high char yield and bio-renewability of lignin. It is hypothesized that overcoming lignin heterogeneity could improve the mechanical properties of the lignin-based carbon fiber. Li et al. developed a non-solvent fractionation technique to reduce lignin heterogeneity and increase lignin-based carbon fiber performance [71]. Their results showed that the high content of β-O-4 inter linkages in a high-molecular-weight lignin fraction improved the miscibility of lignin macromolecules with the guest PAN molecules, enhanced the formation of a crystallite structure in carbon fibers and eventually boosted the mechanical performance of the final carbon fiber product. Without the addition of PAN, however, it was reported that high severity organosolv lignin with fewer impurities, low molecular weight, more condensed structure, more phenolic OH groups and less aliphatic OH groups demonstrated better performance in the melt spinning and thermo-stabilization process, resulting in carbon fibers with higher tensile strength and modulus [75]. Besides adjusting the structure of lignin, the addition of additives such as poly(ethylene oxide) (PEO) into lignin can also enhance the properties of the final carbon fibers [76]. It is suggested that the addition of PEO anisotropically directs the self-assembly of lignin by lengthening the cylindrical building blocks that making larger global aggregates. Liu et al. synthesized a lignin-based carbon fiber with a PAN sheath and a PAN/lignin core via a bi-component gel spinning technique and showed that the obtained bi-component fibers exhibited a comparable tensile property to that of the PAN fiber [77]. Dai et al. showed that iodine treatment effectively enhanced the rigidity of the lignin chain and as a result increased the tensile strength of lignin-based carbon fibers [78].

Compared to traditional PAN-based carbon fiber, lignin-based carbon fibers tend to have relatively low conductivity as a result of their poor graphitic structure, thus limiting their use in bio-electrochemical applications such as photovoltaic cells, batteries and electrochemical sensors. Graphene oxide (GO) has been used to mix with cellulosic materials to alter the morphology of their hydrothermal carbonization product to make the carbon product more conductive [79,80]. Similarly, GO liquid crystal could be added into lignin-based carbon fibers as a templating agent to direct the ordering of lignin molecules during the wet-spinning process and accelerate the formation of a graphitic structure even at low carbonization temperatures, resulting in highly conductive carbon fibers [81]. Newcomb et al. showed that the addition of 0.5 to 1 wt% carbon nanotubes (CNT) resulted in around a 25% increase in electrical conductivity and up to 100% increase in the thermal conductivity of the PAN-based carbon fiber [82]. Therefore, it is reasonable to expect that the same conductivity enhancement would be observed for the lignin-based carbon fiber as well, although a recent study indicated that the PAN/lignin/CNT carbon fibers actually had lower mechanical properties as compared to PAN or PAN/lignin-based carbon fibers [82].

The chemical structure changes of lignin during the carbon fiber production process (pelletizing, melt spinning, stabilization and carbonization) have been monitored by NMR and FTIR and major lignin reactions during the complete process line were subsequently proposed [73,83,84]. The formation of carboxylic acids and ketone was observed during the pelletizing process of lignin [73]. Sun et al. showed that rheological treatment of lignin at 170 °C induced its repolymerization, accompanied by an increase in condensed linkages, molecular weight and viscosities, while rheology testing at 190 °C resulted in a decrease in lignin aliphatic and phenolic hydroxyl groups, β-O-aryl ether linkages, molecular weights and viscosity values [84]. Four major chemical reactions were detectable during the stabilization process: formation of ketones, carboxylic acid, autoxidation of aldehydes and formation of cross-linkages. Finally, the graphitic structure can be postulated in the carbonized fiber [73].

## 5. Lignin and Its Derivatives for Heavy Metal Adsorbent

The demand for clean water is likely to keep increasing, driven by increased industrial/agriculture activities and dramatic population and economic growth. Because freshwater on earth is a limited resource, this demand is likely to be addressed by developing promising water purification methodologies. Among water purification technologies (ion exchange, membrane, coagulation, flocculation, chemical precipitation, biological treatment, etc.), adsorption is widely considered as an effective way to remove pollutants such as heavy metal ions from aqueous solutions. While several organic and inorganic materials, including zeolite [85], activated carbon [86], graphene oxide [87] and carbon nanotubes [88], have been proposed and tested for their ability to remove different pollutants, the development of a low-cost renewable green bio-adsorbent has become a topic of great interest but remains quite challenging. Recently, great efforts have been made to search for cost-effective adsorbents derived from biomass components. Because of its versatile functionality, lignin can be directly incorporated into a polymer matrix or chemically modified to serve as a heavy metal ion sorbent.

Lignin is generally hydrophobic, but it also contains various hydrophilic functional groups such as carboxyl and hydroxyl groups which account for the metallic ion adsorption performance. Thus, as-obtained lignin without further chemical modification could be directly used as heavy metal adsorbents. Todorciuc et al. investigated the adsorption of Cu^2+^ onto wheat straw alkaline lignin, and the results suggest that the Cu ions are mainly retained through an ion-exchange mechanism, with maximum adsorption of 26.0 mg/g [89]. Similarly, effective adsorption of toxic heavy metal ions such as Cr^6+^, Pb^2+^ and Cd^2+^ onto alkaline lignin were also reported elsewhere [90,91]. Besides alkaline lignin, organosolv lignin and lignosulfonate are also reported as heavy metal adsorbents. Harmita et al. compared the adsorption capability of different organosolv lignins toward Cd^2+^ and Cu^2+^ with Kraft lignin, and results indicated that the sorption capacity varied in the following order: softwood organosolv lignin < hardwood organosolv lignin < hardwood Kraft lignin < softwood Kraft lignin [92]. Raw lignin could be also directly incorporated into a polymeric matrix to serve as a pollutant adsorbent as well. A chitin/lignin hybrid material was synthesized as an effective adsorbent for the removal of hazardous metals such as Ni^2+^, Cu^2+^, Zn^2+^ and Pb^2+^ from model solutions as well as real industrial wastewaters [93]. Nair et al. also reported a novel chitosan-lignin composite for effective adsorption of metal ions from wastewater [94]. Song et al. prepared a novel magnetic lignin composite with Fe_3_O_4_ and diethylenetriamine, and the adsorption of Cr^6+^ onto this composite was investigated [95]. An eco-friendly nanocomposite based on lignin grafted carbon nanotubes was prepared as a new type of adsorbent for water remediation; the adsorption results showed that the as-obtained composite exhibited an excellent adsorption capability for Pb^2+^ with 251mg/g [96].

As mentioned above, lignin could be chemically modified to tailor its specific physicochemical properties and by doing so it will create a more reactive site, thus improving its adsorbent behavior. Lignin amination refers to a process that introduces an amine group into the lignin structure. Aminated lignin has been used to remove heavy metal ions such as As^5+^, Cd^2+^ and Cu^2+^ from polluted water [97,98,99]. Li et al. showed that the adsorption capacity of aminated lignin for Pb^2+^ could be controlled by varying the chain length of the alkyl group, and the amount of Pb^2+^ adsorbed by the lignin increased by ~105% as the carbon chain length increased from C2 to C4 [100]. This could be explained by the inductive effect of the alkyl groups. To increase the adsorption affinity toward heavy metal ions, lignin could be also modified with sulfur-containing functional groups as sulfur is a soft base that has a strong affinity to many heavy metal ions. Jin et al. synthesized a 1,2,4-triazole-3-thiol modified lignin-based adsorbent (LBA) for Cd(II) adsorption, and the Langmuir models indicated that LBA exhibited an adsorption capacity of 87.4 mg/g that was around nine times that of the unmodified lignin [101]. Zhou et al. reported a mercapto-functionalized alkali lignin for the adsorption of Hg (II) with the saturated adsorption amounts of 101.2 mg/g [102]. Ogunsile et al. reported a maximum adsorption amount of 15.87 mg/g at 55 °C for Pb^2+^ by a sulfonated resinified lignin [103]. Liu et al. synthesized carboxymethyl lignin nanospheres by a two-step method using microwave irradiation and antisolvent for heavy metal ion adsorption, and the maximum adsorption capacity for Pb^2+^ was found to be 333.3 mg/g, which is significantly higher than other lignin-based adsorbents [104]. Oxidative modification of lignin also could enhance its adsorption behavior due to the greater amount of carboxyl groups presented. For example, organosolv lignin was subjected to oxidation by polyoxometalate (POM), aiming to increase the oxygen-containing adsorption active sites for heavy metal adsorption, and the sorption capacity of the oxidized lignin toward Cd^2+^ and Pb^2+^ was increased threefold and twofold, respectively [105]. Bi-functionalized lignin could be also synthesized and applied as a heavy metal adsorbent. Ge et al. prepared a bio-functionalized lignin via Mannich reaction and sulfomethylation that showed maximum adsorption amounts of 0.71 and 0.26 mmol/g for Cu^2+^ and Pb^2+^ at pH 6.0 and 25 °C, respectively [106]. Table 2 presents the adsorption capacities of some as-obtained and modified lignin for heavy metals in water. Common isotherm and kinetic models used to describe these adsorption processes include Langmuir, Freundlich isotherm and pseudo-first-order and pseudo-second-order kinetic models. A quick literature survey showed that the majority of these adsorption processes conformed to the pseudo-second-order kinetic model and Langmuir isotherm model, suggesting that these adsorption behaviors are dominated and controlled by a chemical monolayer type of adsorption.

## 6. Preparation of Lignin Nanoparticles and Their Applications

The ability to engineer material at its nanoscale could help us address significant challenges in numerous fields. Because of its renewability and biodegradability, lignin has become an ideal precursor for developing environmentally friendly nanoscale materials. Lignin nanoparticles, also known as nanolignin or colloidal lignin particles, have received significant interest in recent years from both research and industrial communities, and their production and application represents an important aspect of future lignin valorization. This section mainly describes the main routes to prepare lignin nanoparticles as well as their applications.

**Figure 12:**
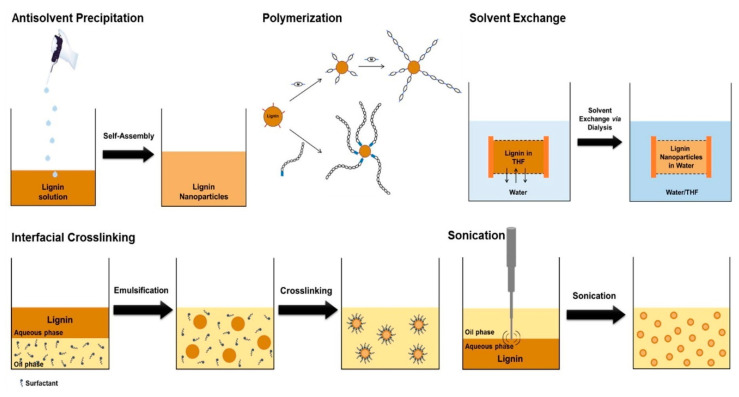
Schematic representation of the different methodologies used to produce lignin nanoparticles. Reprinted from [120] with permission from Elsevier © 2020.

**Figure 13:**
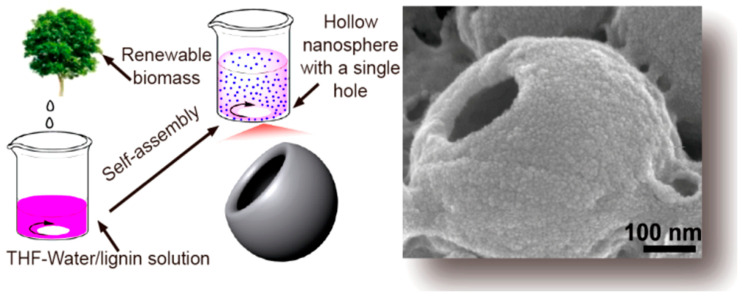
Preparation of renewable lignin hollow nanospheres with a single hole by self-assembly. Reprinted from [117] with permission from American Chemical Society © 2020.

Nanolignin could be prepared via precipitation technology [108], ultra-sonication [109], atomization [110], high shear homogenization [111] and continuous solvent exchange/dialysis for various applications [112]. The techniques used to prepare lignin nanoparticles could be also divided into wet and dry methods [113]. Wet particles are generally produced via various precipitation methods which involve a process of dissolving lignin in organic solvent and a process of precipitating in an anti-solvent (typically water). Lignin nanoparticles obtained in this way have a tendency to form spherical particles that minimize the surface area in contact with the non-solvent phase. On the other hand, dry lignin particles are typically prepared via well-controlled drying of dilute lignin solutions. Figure 12 illustrates a schematic representation of different methodologies to produce lignin nanoparticles. Zhang et al. prepared corncob lignin nanoparticles by adding sodium acetate buffer as the antisolvent to the DMSO/lignin colloidal dispersion [114]. The diameters of the particles were affected by the amount of buffer addition, buffer concentration and initial lignin concentration, and for the first time, the antioxidant activity of the lignin was found to depend on the diameter of the nanoparticles, where smaller particles showed higher activity. Agustin et al. reported a rapid green way to prepare highly charged and spherical lignin nanoparticles from black liquor by combing acid precipitation and ultra-sonication [115]. Liu et al. developed a sequential organosolv fragmentation approach using ethanol plus different-stage catalysts (sulfuric acid, formic acid, sodium hydroxide) to selectively dissolve lignin, and with the help of acid precipitation, a series of lignin nanoparticles with tailored chemical structure and reactivity was prepared [116]. These spherical nanoparticles have diameters ranging from 132 to 1099 nm depending on the used acid catalysts, and their polydispersity index and zeta potential were less than 0.08 and −50 mV, respectively, suggesting the relatively good uniformity and stability of the nanoparticles. While the majority of the lignin nanoparticles reported in the literature have spherical shapes, lignin hollow nanospheres with a single hole could be also prepared by dissolving lignin in tetrahydrofuran followed by dropping DI water into the lignin solution (Figure 13) [117]. The nanospheres of these lignin nanoparticles exhibited a hollow structure due to the effect of THF on the self-assembly behavior. The hollow nanospheres have relatively hydrophilic internal surfaces but hydrophobic external surfaces, corresponding to the hydrophilic aggregated lignin molecules on the internal surface and the hydrophobic lignin membrane formed at the interface between water and THF, respectively. When using the solvent-antisolvent precipitation method to prepare lignin nanoparticles, the size of the particle was reported to be correlated with the molecular weight and chemical structure of lignin. For example, it was reported that lignin fractions with high molecular weight, low content of OH groups and high S/G ratio resulted in weak lignin–water interactions and a high degree of aggregation, thereby resulting in a small size of nanoparticles [118]. Although lignin particles are generally considered non-toxic by in vitro or in vivo toxicological assessment, the current production of lignin nanoparticles often requires the use of organic solvents and thus can generate toxic effects on the environment [113]. In addition, some of the processes also use expensive materials. Moving forward, the utilization of green or renewable solvents and the application of eco-friendly technologies for synthesizing lignin nanoparticles that comply with the green chemistry principle is essential [119].

Applications of lignin nanoparticles in UV protection [121], antibacterial [122], mineral flotation [123], pickering emulsions [108], cosmetics [109], anticorrosion [124] and drug carriers have also been reported in the literature [112]. Wang et al. prepared lignin nanoparticles via solvent exchange combined with the ultrasound process, and the UV adsorbing ability of these lignin nanoparticles was subsequently tested [109]. Results showed that the addition of lignin nanoparticles improved the sunscreen performance of the chemical cream, and the size of nanoparticles played an important role. Dai et al. developed a novel lignin nanoparticle platform to load bioactive molecule resveratrol (RSV) and Fe_3_O_4_ magnetic nanoparticles for drug delivery, and results showed that the nanoparticle system greatly improved the stability, accumulation and anticancer efficacy of RSV in comparison to free drugs [125]. The lignin nanoparticles were prepared via a self-assembly technique by adding water to a methanol solution of alkali lignin.

Nanolignin can be also incorporated into bio-composites to improve their thermal stability, reduce water sensitivity and increase their mechanical performance due to the large number of functional groups present in lignin. It was reported that nanoparticulated lignin could be added into a poly(vinyl alcohol) (PVA) film, simultaneously acting as a strong UV absorber and mechanical enhancer without significantly affecting the transparency of the film [121]. Similarly, Tian prepared lignin nanoparticles via self-assembling of a deep eutectic solvent and ethanol extracted lignin, and when incorporated into a polymeric matrix such as PVA, these lignin nanoparticles products displayed great potential to formulate a transparent composite film with additional UV shielding efficacy, antioxidant functionalities and increased mechanical and thermal performance [126]. Furthermore, the addition of lignin nanoparticles into bio-composites also increased the antimicrobial ability of the materials [127].

## 7. Conclusions

In the current biorefinery and pulping paper industry, substantial amounts of technical lignin streams are being generated on a daily basis. Despite promising high-value opportunities, lignin is still significantly underutilized and is usually burned as a low-value fuel. Overall, lignin could be either depolymerized to low molecular weight chemicals and liquid fuels or utilized in value-added materials, which seeks to utilize lignin as a building block to synthesize value-added polymeric materials. It has been well established that lignin could be used as a partial replacement for phenol in phenol-formaldehyde resins, and ongoing studies are being directed toward utilizing lignin in other areas such as polyurethane, epoxy resins, carbon fibers and macro- and nano-composites. Moving forward, the replacement of conventional petroleum-based polymer sources with lignin-based materials requires significant process improvements and specific expertise from multiple areas across different disciplines. Although the various functionalities of lignin macromolecules offer great opportunities for chemical modifications, the direct use of lignin without chemical treatment represents an effective way to decrease the cost of the overall process. Techniques to cost-effectively isolate lignin from the plant cell wall and methods to chemically modify lignin without the use of expensive reagents need to be identified. Industrial lignin streams normally have a high level of polydispersity; thus, lignin fractionation technologies to further overcome its heterogeneity have been of great interest. Finally, the development of lignin-based value-added polymeric materials with controlled architecture, such as at nanoscale, is essential to broaden lignin’s applications in smart materials but is still largely underexploited. Given the quick development of biotechnology, process chemistry and engineering, we envision that lignin’s application will significantly increase as a green renewable resource for high-performance value-added polymeric materials.

## Figures and Tables

**Table 1:** Lignin copolymers and their applications.

Lignin	Monomer/Prepolymer	Copolymer	Application	Ref.
Kraft lignin	methyl methacrylate	lignin-PMMA	nanofiber	[46]
Kraft lignin	Styrene	lignin-PS	thermoplastics	[49]
soda lignin	hyperbranched poly(ester-amine-amide)	lignin-HBPEAA	memory polymer	[48]
Kraft lignin	acrylic acid (AA)	lignin-PAA	dispersant, flocculant	[44]
alkali lignin	poly(ethylene glycol) methacrylate (PEGMA)	lignin-PEGMA	UV-blocker	[50]
alkali lignin	ε-caprolatone, L-lactide	lignin-PCLLA	nanofiber	[45]
pyrolytic lignin	L-lactide	lignin-PLA	composites	[51]
Kraft lignin	acrylamide, diallyldimethylammonium chloride	lignin-PAM-PDADMAC	flocculant	[52]
Kraft lignin	ε-caprolatone	lignin-PCL	thermoplastics	[53]
alkali lignin	β-butyrolactone	lignin-PHB	nanofiber	[47]

**Table 2:** Adsorption capacities of as-obtained and modified lignin for heavy metals in water.

Adsorbent	Heavy Metal	Adsorption Capacity (mg/g)	pH	T (°C)	Ref.
Alkaline lignin	Cd (II)	63.6	5	28	[97]
Alkaline lignin	Cu (II)	26.0	6	20	[89]
Organosolv lignin (softwood)	Cu (II)	1.38	5.3	24	[92]
Organosolv lignin (softwood)	Cd (II)	0.92	6.5	24	[92]
Organosolv lignin (hardwood)	Cu (II)	2.56	5.3	24	[92]
Organosolv lignin (hardwood)	Cd (II)	2.06	6.5	24	[92]
Lignin/chitin film	Cu (II)	0.28	3–4	R.T	[107]
Lignin/chitin film	Fe (III)	1.21	3–4	R.T	[107]
Lignin/chitin hybrid material	Ni (II)	70.4	5	25	[93]
Lignin/chitin hybrid material	Cu (II)	75.7	5	25	[93]
Lignin/chitin hybrid material	Zn (II)	82.4	5	25	[93]
Lignin/chitin hybrid material	Pb (II)	91.7	5	25	[93]
Aminated lignin	Cd (II)	43.2	5	28	[97]
Aminated lignin	Cu (II)	37.1	5.5	40	[98]
Aminated lignin	As (V)	62.5	9	25	[99]
Triazole modifed lignin	Cd (II)	87.4	6	25	[101]
Mercapto modified alkali lignin	Hg (II)	101.2	4	20	[102]
Carboxymethyl lignin	Pb (II)	333.3	6	30	[104]

## Notice

This manuscript has been authored by UT-Battelle, LLC, under contract DE-AC05-00OR22725 with the US Department of Energy (DOE). The US government retains and the publisher, by accepting the article for publication, acknowledges that the US government retains a nonexclusive, paid-up, irrevocable, worldwide license to publish or reproduce the published form of this manuscript, or allow others to do so, for US government purposes. DOE will provide public access to these results of federally sponsored research in accordance with the DOE Public Access Plan (http://energy.gov/downloads/doe-public-access-plan).
